# The role of RORα in salivary gland lesions in patients with primary Sjögren’s syndrome

**DOI:** 10.1186/s13075-018-1698-5

**Published:** 2018-09-06

**Authors:** Xiuhong Weng, Yi Liu, Shun Cui, Bo Cheng

**Affiliations:** 1 Department of Stomatology, Zhongnan Hospital of Wuhan University, 169 Donghu Road, Wuhan, 430071 Hubei Province China; 2 Department of Stomatology, Union Hospital, Tongji Medical College, Huazhong University of Science and Technology, 1277 Jiefang Ave, Jianhan District, Wuhan, 430022 Hubei Province China; 3 Department of Rheumatology, Union Hospital, Tongji Medical College, Huazhong University of Science and Technology, 1277 Jiefang Ave, Jianghan District, Wuhan, 430022 Hubei Province China

**Keywords:** Primary Sjögren’s syndrome, RORα, Focus score, Th17 cells, Inverse agonist

## Abstract

**Background:**

The orphan nuclear receptors retinoic acid-related receptor α and γt (RORα and RORγt) are critical in the development of T helper 17 (Th17) cells, and ROR-specific synthetic ligands have proven efficacy in several mouse models of autoimmunity. However, the pathological significance of RORα in primary Sjögren’s syndrome (pSS) remains to be elucidated. The present study was designed to clarify the significance of RORα in the pathogenesis of pSS.

**Methods:**

RORα expression in the labial salivary gland (LSG) was determined by immunohistochemical analysis using a quantitative scoring system in 34 patients with pSS. The correlation between RORα expression in LSGs and the focus score (FS) was determined, and Th17 and IL-17 receptor A (1L-17RA) levels in LSGs were determined. To investigate the effect of RORs and the therapeutic potential of targeting RORs in pSS, we administered SR1001, a selective RORα/γt inverse agonist, to non-obese diabetic (NOD) mice.

**Results:**

The expression of RORα was significantly increased in LSGs of patients with pSS and intensified with disease stage/FS, showing a similar increasing trend with IL-17A and IL-17RA. SR1001 significantly improved salivary gland secretory function and relieved sialadenitis in treated mice.

**Conclusion:**

Our data reveal the importance of RORα in controlling pathologic lymphocytic infiltration of the salivary glands and suggest that RORα may be a druggable target in treating pSS.

**Electronic supplementary material:**

The online version of this article (10.1186/s13075-018-1698-5) contains supplementary material, which is available to authorized users.

## Background

Primary Sjögren’s syndrome (pSS) is a chronic, systemic autoimmune disease characterized by lymphocyte infiltration into exocrine glands, such as salivary and lacrimal glands. The main clinical manifestations include xerostomia, xerophthalmia and several systemic manifestations. In addition, the probability of developing lymphoma is significantly higher (up to 44-fold higher) in patients with pSS than in the normal population [1, 2].

Focal lymphocytic sialadenitis is the characteristic histopathological feature of pSS. Minor salivary glands are more likely to have a focus score (FS), which is an index of inflammation, of at least 1 when evaluated by an expert histopathologist [3]. In addition, high concentrations of anti-Ro/La antibodies (SSA/SSB), anti-nuclear antibody (ANA), rheumatoid factor (RF) and immunoglobulin G (IgG) are detected in the plasma of patients with a FS greater than or equal to 1 [4] . Thus, it has been suggested that the FS may be associated with disease activity. The diagnosis of pSS is based on clinical manifestations and laboratory examination [5]. Immunological studies must include a determination of autoantibodies to the SSA and SSB antigens. Minor salivary gland (MSG) biopsy is highly specific for the diagnosis of SS and is indicated principally in patients who are negative for anti-SSA/SSB antibodies.

The pathogenesis of pSS is not yet clear. Immunohistochemical studies have shown that T helper 17 (Th17) cells are among the infiltrating lymphocytes in the labial salivary glands (LSGs) and lacrimal glands [6–9] . High levels of interleukin-17 (IL-17) and Th17-related cytokines have recently been identified in the salivary glands and plasma of patients with pSS and in mouse models of pSS [6, 8, 9]. Blocking IL-17 can significantly improve salivary gland function and reduce gland inflammation in pSS animal models [10]. Retinoic acid-related orphan receptors (RORs) are transcription factors that participate in the differentiation of inflammatory Th17 cells and cytokine production [11–15]. RORα cooperates with RORγt and nuclear factor κB inhibitor ζ (IκBζ) to enhance IL-17 expression by binding directly to the regulatory region of the IL-17A gene [13, 16]. Although several studies have indicated that Th17-related cytokines may be involved in the development of pSS, there is little information on the pathological importance of RORα in pSS. To address this issue, we used the inverse agonist SR1001, which has been found to obstruct Th17 differentiation by specifically inhibiting RORα [11]. This study aimed to investigate the role of RORα in salivary gland inflammation and function in pSS and to explore its role in the progression of pSS, with the goals of providing a theoretical basis and scientific evidence for key targets in the pathogenesis of pSS and improving the clinical diagnosis, staging, and treatment of pSS.

## Methods

### LSG histology

LSG biopsy specimens were obtained with informed consent from 34 individuals undergoing diagnostic evaluation for sicca symptoms indicative of pSS and diagnosed by American-European Sjögren’s syndrome (SS) consensus criteria. The control group consisted of 12 gender-matched individuals with subjective complaints of dry mouth or eyes but who did not fulfill the criteria for pSS and had no histopathological evidence of pSS. None of the patients had evidence of lymphoma, sarcoidosis, essential mixed cryoglobulinemia, or HIV or hepatitis B or C virus infection at the time of the study. In addition, patients’ medical records were evaluated for clinical and serological parameters, including SSA and SSB antibodies, high erythrocyte sedimentation rate (ESR), and C3/C4 hypocomplementemia.

The inflammatory lesions were graded histologically (histological FS) using the following method proposed by Greenspan: FS 1, a single focus composed of ~ 50 mononuclear cells per 4 mm^2^ tissue. Salivary gland histopathology and rank were evaluated by researchers who were blinded to diagnosis, and at least one tissue section from each salivary gland was examined. Biopsy specimens were fixed, embedded, sectioned (4 μm/section), deparaffinized, rehydrated in alcohol, and stained with H&E.

### Immunofluorescence of human samples

Paraffin sections were placed on silane-coated slides, dewaxed, rehydrated, and heated in citric acid (pH 6.0) buffer for 7 min for antigen retrieval. The sections were incubated with ice-cold blocking solution (PBS containing 5% (vol/vol) donkey serum and 1% (wt/vol) BSA) for 1 h and the primary antibody (RORα, 1:200; Abcam, UK) diluted 1/10 in blocking solution overnight at 4 °C, followed by three washes with Tris-buffered-saline Tween (TBST), and finally incubated with 488 Donkey-Anti-Rabbit IgG (H + L) (1: 400, Jackson Immunoresearch) for 1 h. Nucleus was stained with diamidino-phenyl-indole (DAPI) (Guge, Wuhan). Staining of CD4 (1:100, Biolegend) and IL-17 (1:100, Peprotech) was as herein before. Confocal images were acquired with the same pinhole diameter for each channel.

### Immunohistochemical analysis of human samples

Paraffin sections were placed on silane-coated slides, dewaxed, rehydrated and heated in citric acid (pH 6.0) buffer for antigen retrieval. Endogenous peroxidase activity was blocked with 3% H_2_O_2_ for 15 min. Then, sections were incubated with blocking serum (ZSGB-BIO, Beijing) for 1 h and then with IL-17RA (1:100, Sangon Biotech, Shanghai) primary antibody overnight at 4 °C. After 20-min incubation with biotinylated secondary antibody (ZSGB-BIO, Beijing), staining was developed for 20 min with an Avidin: Biotinylated Enzyme Complex (ZSGB-BIO, Beijing), followed by 3,3-diaminobenzidine (DAB) staining (ZSGB-BIO, Beijing) and counterstaining with Meyer’s hematoxylin for 1 min. Slides were washed in TBST 3 × 5 minutes after each step. Staining of RORγt (1:50, Abcam) was done as herein before.

### Mice

Female NOD/LtJ mice, 4 weeks old (4W) and 8 weeks old (8W) were obtained from Huafukang (Beijing) and were maintained in specific pathogen-free conditions at the Animal Experiment Center of Huazhong University of Science and Technology. NOD mice were injected intraperitoneally (i.p.) twice a day with vehicle or SR1001, which was dissolved in a 50% dimethyl sulphoxide (DMSO) and 50% normal saline (dose 2.5 mg/ml, equal to 25 mg/kg and 1 μl/g body weight). Mice were divided into four groups: 4W mice treated i.p. with SR1001 or vehicle; and 8W mice treated i.p. with SR1001 or vehicle. Each group received SR1001 or vehicle i.p. for 4 weeks. Salivary gland function (measurement of saliva flow rate) and blood glucose (Roche, Swizterland) were evaluated before and after the drug intervention. Body weight was measured every day to monitor any effects of SR1001on growth and development. After 4 weeks, mice were sacrificed, and the isolated salivary glands were H&E stained to observe salivary gland structure. The treatment effect of SR1001 on salivary gland function was measured by saliva volume secreted in 15 min. Mesenteric lymph nodes (MLNs) and cervical lymph nodes (CLNs) were removed, and the ratio of CD4+/IL-17A+ cells was measured by flow cytometry. All experiments were performed according to the Guide for the Care and Use of Laboratory Animals at Tongji Medical College.

### Measurement of stimulated saliva flow

Each NOD mouse was first anesthetized with an i.p. injection of pentobarbital (25 mg/kg/body weight, Sigma-Aldrich, USA) and a subsequent i.p. injection of pilocarpine (1.0 mg/kg/body weight, Leqi, Wuhan). At 1 min after injection of the secretagogue, saliva was collected from the oral cavity using a 200-μl micropipette for 15 min at room temperature. The volume (μl) of each saliva sample was measured and expressed relative to body weight (grams). Baseline saliva flow rates were measured 3 days before the drug injection, and final saliva flow rates in each group were measured the end of the drug injection.

### Flow cytometry

Tissue was cut into small pieces for flow cytometric analysis of MLNs and CLNs cells. T cells were cultured for Th17 differentiation as previously described [17]. Staining for CD4 (1:100, BioLegend) expression was performed for 20 min using a mixture of antibodies. Intracellular staining for IL-17A (1:100, BioLegend) was performed after fixation and permeabilization according to the protocol supplied by the manufacturer (BD Biosciences). Samples were analyzed with a FACS Calibur Flow Cytometer (BD Biosciences), and data were analyzed with FlowJo software (Tree Star, Ashland, OR, USA).

### Western blot

Minor saliva gland proteins were isolated, and western blotting was performed as follows. Proteins were extracted in the radio-immunoprecipitation (RIPA)-cocktail buffer and centrifuged. The supernatant was collected as the RIPA soluble fraction, and the pellet was washed with RIPA buffer, centrifuged and boiled with Laemmli buffer. The concentration of extracted proteins was measured using a BCA protein assay kit (Byotime, Shanghai). The following primary antibodies were used: rabbit anti-RORα (1:800, ab60134, Abcam), rabbit anti-IL-17RA (1:1000, Sangon Biotech, Shanghai), anti-RORγt (1:500, Invitrogen 14–6988-80), and mouse anti-GAPDH (1:5000, Santa Cruz Biotechnology, USA). Protein samples were separated on a 4–12% SDS-PAGE gel and electro-transferred onto a polyvinylidene fluoride (PVDF) membrane for 2 h. Membranes were blocked in blocking solution (TBST containing 5% (wt/vol) milk serum and 1% (wt/vol) BSA) for 1 h at room temperature and then incubated with primary antibodies overnight at 4 °C. Next, membranes were washed in TBST and then incubated with secondary HRP-conjugated anti-mouse (1:5000, Aspen, Wuhan) or anti-rabbit (1:5000, Aspen, Wuhan) antibodies for 1 h at room temperature. The signal was detected with enhanced chemiluminescence (ECL) Western Blotting Substrate (Thermo Scientific Pierce, P180196).

### RNA extraction and real-time quantification PCR

Total RNA was obtained from frozen human labial gland specimens from patients with and without pSS using Trizol reagent according to the protocol supplied by the manufacturer (TaKaRa, Japan). Complementary DNA (Cdna) was synthesized from RNA using PrimeScript RT reagent Kit with gDNA Eraser (TaKaRa, Japan). Real-time PCR for H-RORγt and RORα were performed on StepOne Real-Time (Life Technologies) using the SYBR® Premix Ex Taq kit (TaKaRa, Japan), and H-GAPDH as normalization control. We used the 2^-ΔΔCT^ method for data analysis. Each sample was tested in triplicate, and tests were replicated twice. The primer sets for RT-PCR can be found in Table 1.

**Table 1:** Primer sets for RT-PCR

Primer		Primer sequence	Product length (bp)
H-GAPDH	Forward	5′ - CATCATCCCTGCCTCTACTGG-3′	259
	reverse	5′ - GTGGGTGTCGCTGTTGAAGTC-3′	
H-RORγt	Forward	5′ - TGGAAGTGGTGCTGGTTAGG-3′	203
	reverse	5′ - GAGAACAAGGGCTGTGTAGAGG-3′	
H-RORα	Forward	5′ - CCGTAGGGATGTCTCGAGATG-3′	211
	reverse	5′ -TCAATGTAGTTACTGAGGTCGTCG-3′	

### Statistical analysis

Data were analyzed by the two-tailed Student’s t test using Graphpad Prism. Data are presented as the mean or mean ± SEM. *P* values are denoted as follows: *P < 0.05, **P < 0.01, ***P < 0.001.

## Results

### Patients

RORα expression in LSGs was determined in 46 patients with sicca syndrome who were referred to our department. Thirty-four patients met the American-European Consensus Group criteria for pSS. The remaining 12 patients were classified as having non-SS sicca syndrome. The characteristics of these patients are shown in Table 2). The mean (± SEM) age of the patients with pSS and those with non-pSS was 34.32 ± 2.225 years and 27.25 ± 7.186 years, respectively. All patients were female.

**Table 2:** Characteristics of patients with and without pSS

Variable	pSS (*n* = 34)	NC (*n* = 12)
Age, mean (SEM)	34.32 (2.225)	27.25 (7.186)
Sex (female/male)	34/0	12/0
Focus score ≥ 1, *n* (%)	29 (85.3)	0 (0)
Anti-Ro/SSA, *n* (%)	30 (88.24)	0 (0)
Anti-La/SSB, *n* (%)	19 (55.88)	0 (0)
Both Anti-Ro/SSA and Anti-La/SSB, *n* (%)	19 (55.88)	0 (0)
ANA, *n* (%)	32 (94.12)	0 (0)
ESR↑, *n* (%)	17 (50)	0 (0)
IgG > 15.9, *n* (%)	24 (70.59)	0 (0)
RF > 15.9KU/I, *n* (%)	15 (44.12)	0 (0)

*pSS* primary Sjögren’s syndrome, *NC* normal controls, *Anti-Ro/SSA/SSB* anti-Sjögren’s-syndrome-related antigen antibody A/B, *ANA* anti-nuclear antibodies, *ESR* erythrocyte sedimentation rate, *RF* rheumatoid factor

#### Increased RORα expression in LSGs of patients with pSS

**Fig. 1:**
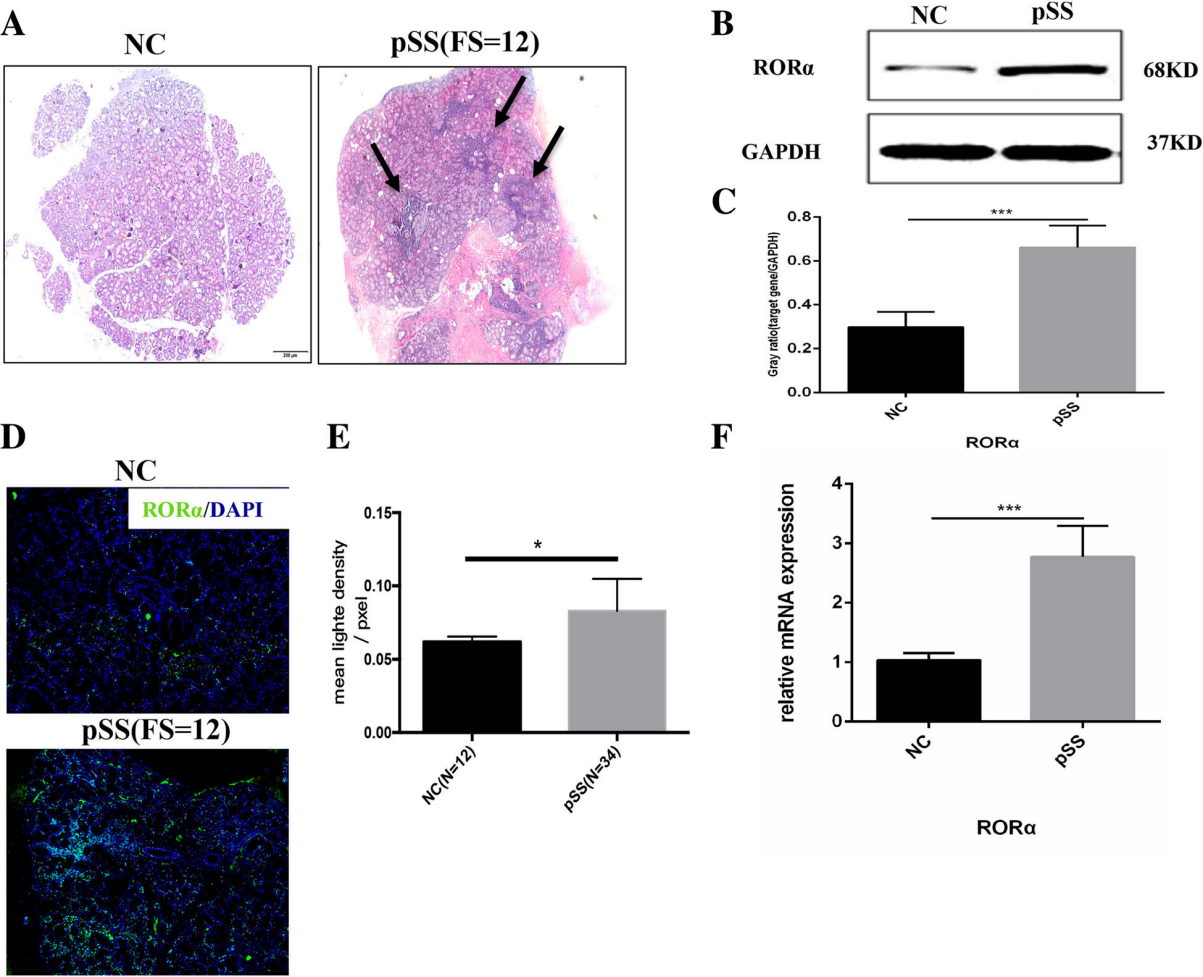
Higher RORα expression in the labial salivary glands (LSGs) of patients with primary Sjögren’s syndrome (pSS). **a** Pathological analysis of LSG sections. Left, histologic image of normal LSGs from non-SS patients (normal control (NC)). Right, typical histologic image of LSGs from patients with pSS, including acinus atrophy and local lymphocytic sialadenitis. Arrowheads indicate lymphocyte infiltration in connective tissue among glandular lobules and ducts. Scale bars = 200 μm. **b** Western blot showed that expression of RORα (68 kD) was significantly higher in whole LSGs of patients with pSS (*n* = 6) compared to NC (*n* = 4). **c** Gray-scale analysis. Data were normalized for glyceraldehyde-3-phosphate dehydrogenase (GAPDH). Data are mean ± SEM. ***p < 0.001. **d** Expression of RORα (green) was higher in LSGs of patients with pSS shown by indirect immunofluorescence analysis. Nuclei stained by diamidino-phenyl-indole (DAPI) (blue). Scale bars = 100 μm. **e** normalized fold difference in RORα expression between pSS (*n* = 34) and NC (*n* = 12) analyzed by densitometry. ^*^p < 0.05. **f** Relative expression of RORα mRNA in whole LSGs of patients with pSS (*n* = 6) compared to NC (*n* = 4) are normalized for GAPDH mRNA and plotted as fold change over control. Data are mean ± SEM. ***p < 0.001

**Fig. 2:**
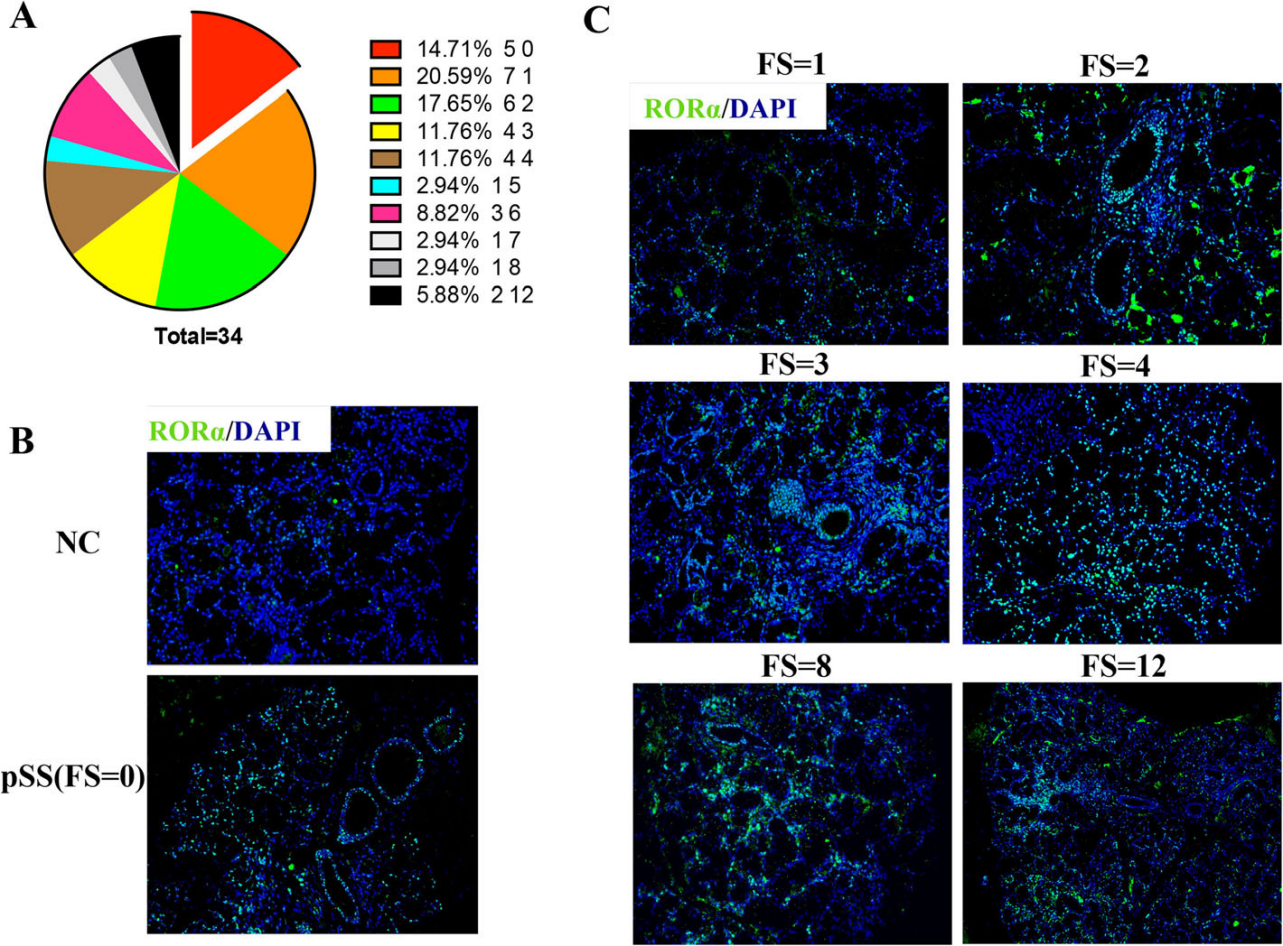
RORα expression in the labial salivary glands (LSGs) of patients with primary Sjögren’s syndrome (pSS) increased with increasing focus score (FS). **a** Focal index of all patients with pSS (*n* = 34) and the statistical analysis: 85.3% (*n* = 29) of pSS in our study had obvious local lymphocytic sialadenitis (FS > =1). **b** Expression of RORα expression (green) in LSG sections from patients with pSS with a FS of 0 (*n* = 5, lower panel) was still higher than in patients without pSS (normal control (NC) (*n* = 10, upper panel) assessed by indirect immunofluorescence. **c** RORα expression in patients with pSS with a different FS (*n* = 29) assessed by indirect immunofluorescence analysis suggested that the RORα expression in the LSGs in pSS increased with increasing FS. Diamidino-phenyl-indole (DAPI) staining for nuclei (blue). Scale bars = 100 μm

Typical histologic characteristics of LSGs from patients with pSS were acinus atrophy and local lymphocytic sialadenitis (Fig. 1a). We detected the expression of RORα in LSGs by different methods, and all indicated that RORα expression was significantly up-regulated in the LSGs of patients with pSS (Fig. 1b–d). RORα-expressing cells were observed in the LSGs of nearly all patients with sicca syndrome, even in those without obvious local lymphocytic sialadenitis (FS = 0) (Fig. 2b).

#### RORα expression increased with increasing FS

RORα expression was monitored in the LSGs of pSS patients with different FS and this indicated that RORα expression increased with increasing FS (Fig. 2c), which might suggest its participation in the pathogenesis of pSS. LSGs from all pSS patients with obvious focal lymphocytic accumulation (FS ≥ 1) had a high number of RORα-positive cells localized predominantly around the ducts (Fig. 2b, c). For RORα protein expression in LSG biopsy specimens intensified with disease stage/FS, there might be indicative of a correlation between RORα up-regulation and pathological manifestations in LSGs of pSS, but it remained unclear whether this deposition correlated with known markers of pSS, such as anti-Ro (SSA) and anti-La (SSB) antibodies, high erythrocyte sedimentation rate (ESR), or C3/C4.

#### The ratio of CD4+/IL-17A+ cells in the salivary gland was higher in patients with pSS than in control individuals

**Fig. 3:**
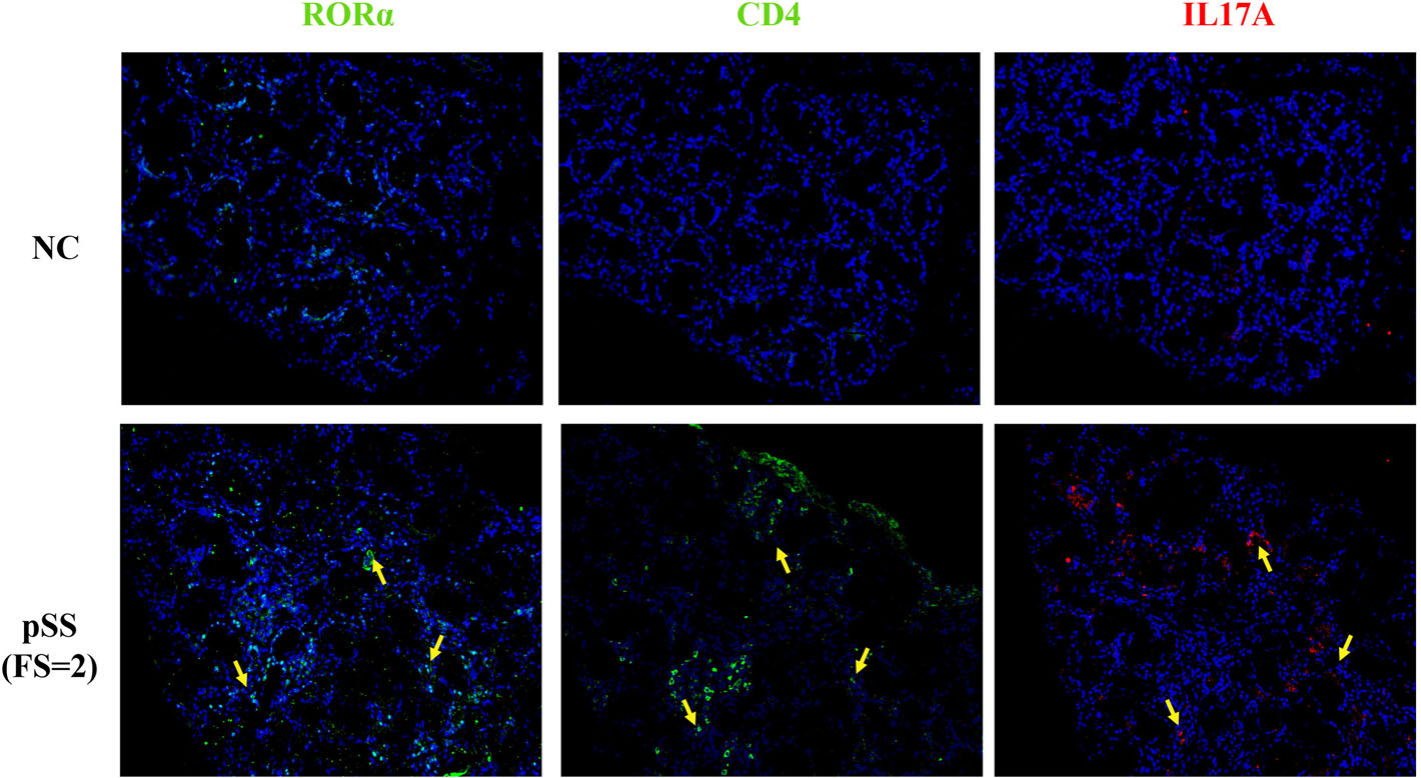
Co-expression of RORα with CD4 and IL-17A in the labial salivary glands (LSGs). Indirect immunofluorescence analysis was used to assess RORα (green), CD4 (green) and IL-17A (red) expression and diamidino-phenyl-indole (DAPI) staining for nuclei (blue) in the LSG. Results are representative of 30 patients with primary Sjögren’s syndrome (pSS) (lower lane) and 10 normal control individuals (NC) (upper lane). The micrographs in the lower panel showed RORα-positive T helper 17 (Th17) cells in the LSGs of patients with pSS (focus score (FS) = 2), whereas the upper panel only shows some RORα-positive cells in the LSGs of NC. It indicated RORα-positive Th17 cells in the periductal tissue of LSGs of patients with pSS. Serial sections of the LSG from the same patient, and yellow arrows (lower lane) in the same direction indicated the same cell in three serial sections. Scale bars = 100 μm

RORα-positive Th17 cells were detected in LSGs from patients with pSS (Fig. 3), indicating that RORα regulates Th17 cell differentiation and is involved in the pathogenesis of pSS. IL-17 was secreted in large amounts by inflammatory cells from patients with pSS, which can facilitate pro-inflammatory responses and tissue destruction. An elevated number of IL-17-producing cells in patients with pSS was correlated with increased glandular inflammation, as indicated by an increased FS in the LSGs. A large number of IL-17-producing cells was observed in the vicinity of LSG lymphocytic infiltrates and in the interstitium.

#### IL-17RA expression was significantly higher in salivary gland tissues from patients with pSS than in that from control individuals

**Fig. 4:**
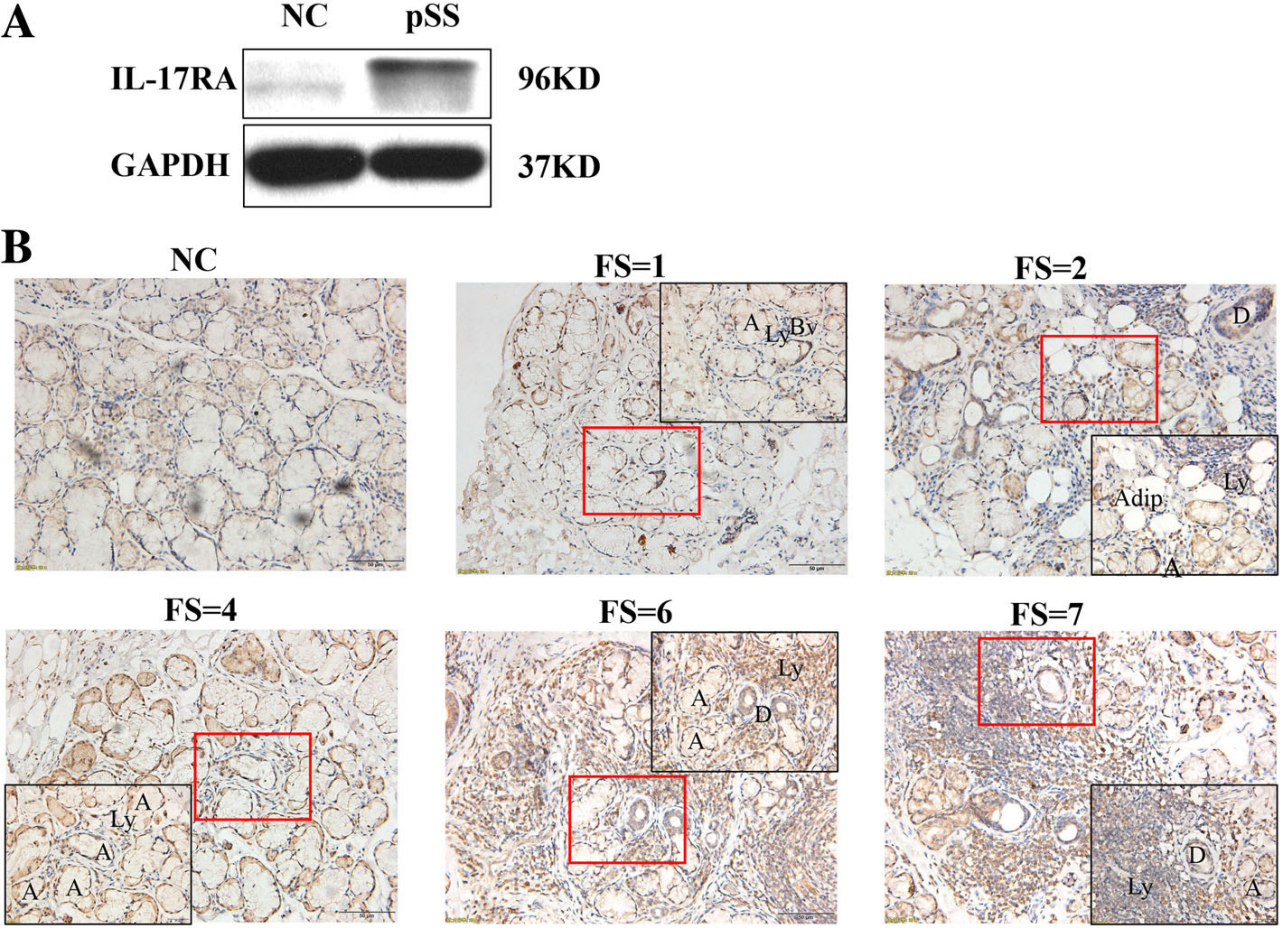
Higher expression of IL-17RA in the labial salivary glands (LSGs) of patients with primary Sjögren’s syndrome (pSS). **a** Protein expression level of IL-17R (96 kD) in LSGs. Expression of IL-17R in tissue with pSS (*n* = 6) was higher compared with normal control (NC) (*n* = 4). **b** Immunohistochemical analysis of local IL-17R in patients with pSS (*n* = 34) and NC (*n* = 10) LSGs. IL-17R-positive cells are significantly more numerous in the LSGs of patients with pSS compared with NC, along with an upward trend as the focus score (FS) increased. Scale bars = 50 μm; insets, × 2 magnification. Abbreviations: A, acinus; Adip, adipocytes; Bv, blood vessel; D, ductus; Ly, lymphocyte infiltration

Immunohistochemical staining revealed substantial expression of IL-17RA in the LSGs from pSS patients (Fig. 4b), not only seen in infiltrated lymphocytes but also in acinus and ductal epithelial cells. Furthermore, high expression of IL-17RA was detected by western blot (Fig. 4a). In addition, IL-17RA expression also tended to increase with increasing disease stage/FS as did RORα. The number of IL-17+ cells may be inflated, because the widespread distribution of IL-17RA may confound quantification. It is not possible to dissociate cells that produce IL-17 from those that bind/respond to it, and multiple cell types may be responsible for secreting IL-17. Because of the difficulty in accurately quantifying IL-17RA-expressing populations by immunohistochemical analysis, we assessed IL-17RA by western blot of proteins extracted from whole LSGs from patients with pSS and control individuals (Fig. 4a). We observed higher IL-17RA expression in the LSGs of patients with pSS (*n* = 34) compared with control individuals (*n* = 12).

#### Synthetic RORα inverse agonist improved salivary gland function and alleviated lymphocytic infiltration of salivary glands

**Fig. 5:**
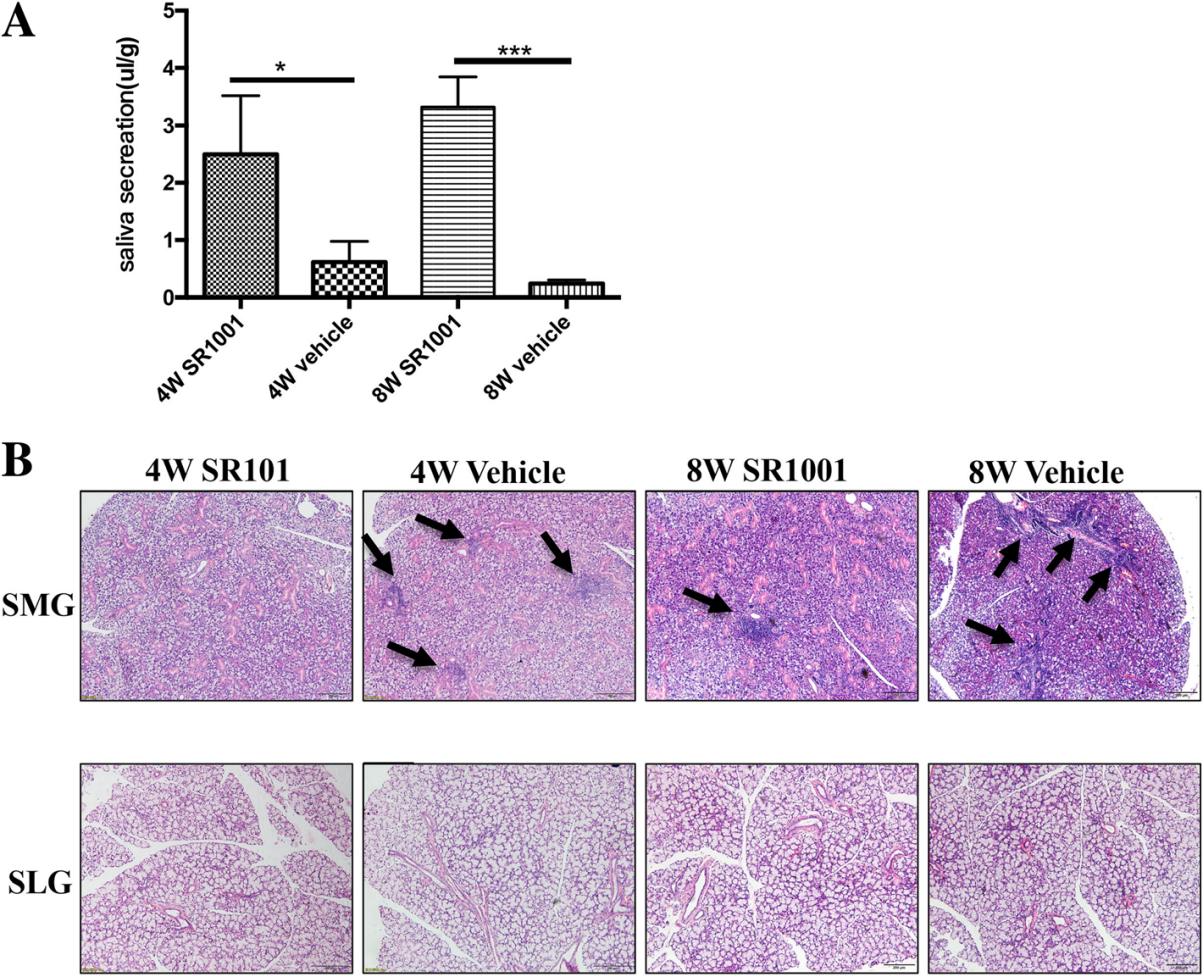
SR1001 improved salivary gland function and alleviated lymphocytic infiltration of salivary glands in non-obese diabetic (NOD) mice. **a** SR1001-treated NOD mice exhibited normal salivary secretion, while significantly decreased saliva flow rate was observed in the both vehicle-treated 4-week-old (4W) and 8-week-old (8W) groups (*n* = 5 for each group); data represent means ± SEM (μl/g): ^*^p < 0.05, ***p < 0.001. **b** Histological evaluation of glandular destruction in NOD mice was performed on tissue sections of submandibular glands (SMGs) (upper lane) and sublingual glands (SLGs) (lower lane) by H&E staining (*n* = 5 for each group). Arrowheads indicate inflammatory infiltrate foci. The upper micrographs showed fewer lymphocyte infiltration foci in SMGs of 4W SR1001-treated NOD mice at both 4 and 8 weeks of age compared to vehicle groups, and older NOD mice (8W) had mild inflammatory infiltration. The lower panel indicates no obvious sialadenitis in SLGs of NOD mice (*n* = 5 for each group). Scale bars = 100 μm

To verify the secretory function of salivary glands, saliva was collected from NOD mice. Significantly more saliva was collected from SR1001-treated NOD mice than from littermate vehicle controls in both the 4W and 8W groups (Fig. 5a), but the difference between the 8W groups was more significant (Fig. 5a). Then, NOD mice were sacrificed for histological analysis to determine the effect of SR1001 on sialadenitis development. More infiltrating mononuclear cells were detected in the SMGs of vehicle-treated NOD mice. SR1001 treatment significantly reduced lymphocytic infiltration of SMGs compared with littermate vehicle controls in both the 4W and 8W groups (Fig. 5b). This result might suggest that pharmacological inhibition of RORα effectively alleviated salivary gland destruction and improved salivary gland function.

**Fig. 6:**
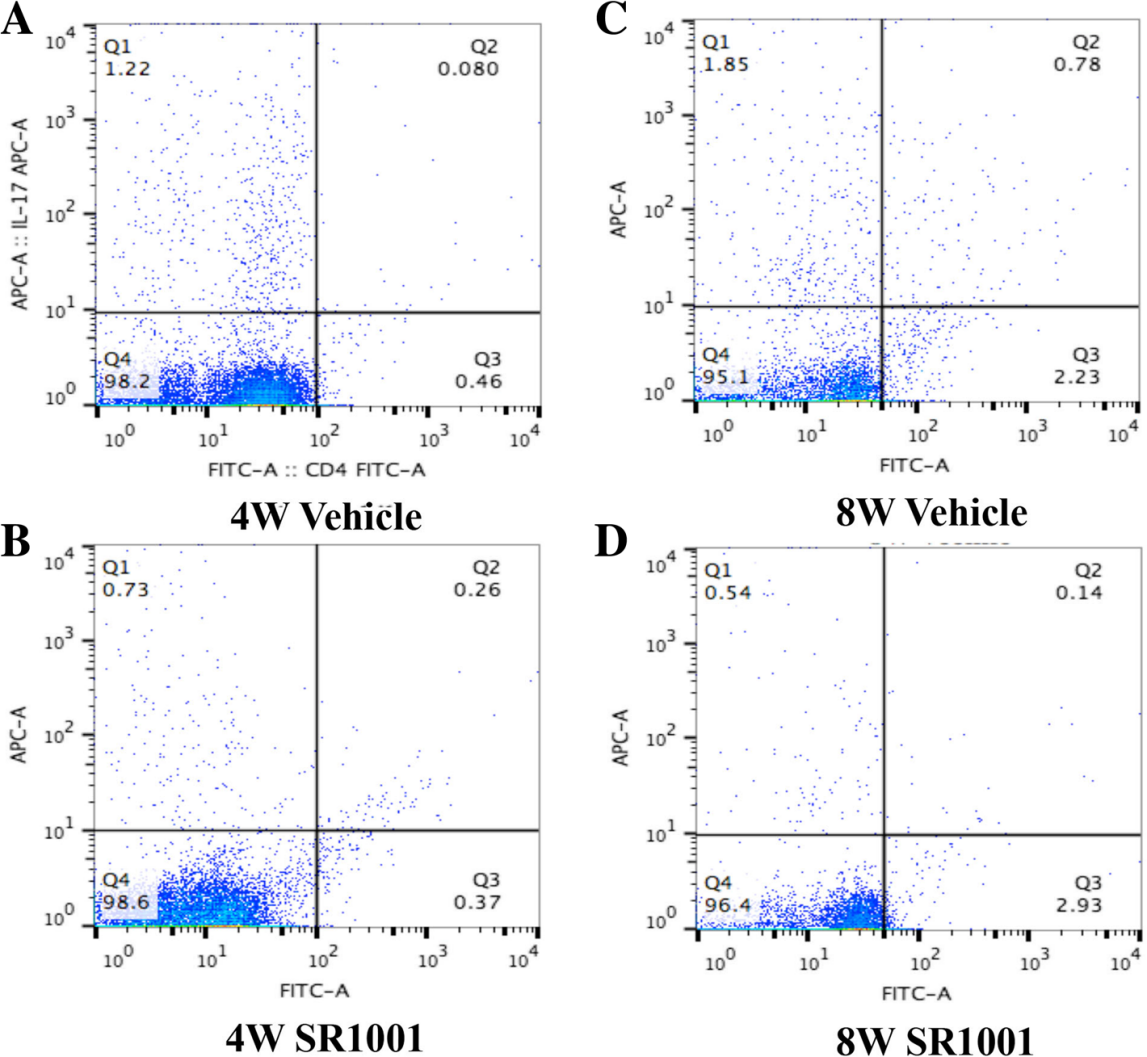
Phenotypic analysis of T cells in mesenteric lymph nodes (MLNs) and cervical lymph nodes (CLNs) by flow cytometry after 4-week treatment with SR1001 or vehicle. CD4+ and IL-17+ cells in MLNs and CLNs were detected by flow cytometry after stimulation with phorbol-12-myristate-13-acetate (PMA) and ionomycin for 6 h (*n* = 3 for each group). CD4+ and IL-17+ double positive cells (T helper 17(Th17)) were decreased in MLNs and CLNs of non-obese diabetic (NOD) mice after 4-week SR1001treatment at both 4 weeks (4W) and 8 weeks (8W) of age (**b**, **d**) compared to vehicle groups (**a**, **c**)

Moreover, flow cytometry analysis of CD4+ T cells in MLNs and CLNs from different-aged NOD mice that received different treatments after stimulation with phorbol-12-myristate-13-acetate (PMA) and ionomycin for 6 h was performed to determine the effect of SR1001 on Th17 cell differentiation (Fig. 6). It indicated that SR1001, a synthetic RORα inverse agonist, decreased Th17 differentiation of CD4+ T cells in MLNs and CLNs after 4-week treatment (Fig. 6). We also detected that SR1001 has the ability to regulate blood glucose in NOD mice, especially in mice at 8 weeks of age (Additional file 1B). Meanwhile, systemic treatment of SR1001 had no effect on body weight gain in NOD mice (Additional file 1A).

## Discussion

Dry mouth, dry eyes and rampant caries due to a reduction in exocrine secretions are the main clinical manifestations of pSS, which cause patients considerable inconvenience and pain [18, 19]. As reported in previous studies, most patients with pSS are female [20, 21]. Lymphocytic infiltration of salivary glands and impaired saliva secretion are the main features of pSS. A variety of autoantibodies, such as high ESR, RF, and Ig, can be detected in the serum of patients with pSS. Although there have been great improvements in our knowledge and understanding of pSS, diagnosis, treatment and disease monitoring are still challenging in the clinic.

pSS is known as an autoimmune disease caused by a group of different mechanisms, and immune disorder is important in pSS occurrence and development. Due to the complexity and integrity of the immune system, functional cells involved in immune responses may all be involved in the pathogenesis of pSS, in which the study of T/B cells predominates. The role of Th17 cells in autoimmune diseases has been widely recognized [14, 22]. In fact, lymphocytic infiltration into the exocrine glands of patients with pSS does not contain a single species but includes Th1, Th2, Th17, T regulatory (Treg), and B cells [23–25]. Treg cells are the most important immune-suppressing cells, and it is believed that the imbalance between Th17 cells and Treg cells is crucial in the progression of pSS [26, 27]. Th17 cells comprise a pro-inflammatory cell subset that can promote inflammatory reactions and secrete various pro-inflammatory factors [26]. Both patients with pSS and mouse models harbor Th17 cells and IL-17 in the salivary glands and in serum [28–30]. Furthermore, serum IL-17 levels are closely related to the degree of salivary gland damage [9].

Th17 cells and IL-17 do not directly lead to an inflammatory reaction. The pro-inflammatory effect is mainly due to pro-inflammatory cytokines, such as IL-6 and granulocyte-macrophage colony stimulating factor (GM-CSF), produced in response to IL-17 binding to IL-17RA, which occurs in the salivary glands and causes tissue damage [31, 32]. However, it has been proved that IL-17 was not essential for the development of sialadenitis by examination of IL-17-deficient mice [13]. The levels of IL-17A and IL-17RA can be regulated by each other [33, 34]; IL-17RA could be rapidly down-regulated by IL-17A binding [35], and IL-17RA has some ability to clear local IL-17A, which may reduce inflammation [36]. In the present study, we found Th17 cells and IL-17RA were significantly increased in the LSGs of patients with pSS, and the expression of IL-17RA tended to increase with increasing FS. In addition, we found lymphocytes and part of the glandular and ductal epithelial cells were IL-17RA positive. It may indicate that local increased IL-17 binding to IL-17RA promotes immune effects that cause salivary gland destruction, suggesting that IL-17RA is associated with the progression of pSS.

RORα is one member of the ROR family, which is widely expressed in a variety of tissues and participates in various biological processes, such as immune response, cerebellar development, and biological rhythms [11, 37, 38]. RORs regulate the transcription of downstream target genes in a ligand-independent manner by binding to ROR response elements with co-stimulatory transcription factors [39, 40]. RORγt and RORα are important in the differentiation and maturation of Th17 cells [11, 14, 18]. In this study, we found that RORα expression was significantly higher in the LSGs of patients with pSS than in non-SS LSGs, and it showed a trend of increasing as FS increased. These data suggested that RORα expression is associated with the progression of pSS. These results may be attributed to the effects of RORα on Th17 cells, i.e., increased IL-17 synthesis and secretion, because RORα-positive Th17 cells were detected in the salivary glands of patients with pSS in our study. Approximately 25% of patients diagnosed as having pSS had no obvious evidence of sialadenitis in LSG biopsies, which is consistent with the results of previous studies [3]. Nevertheless, the LSGs in patients with pSS without obvious FS still had extensive, high RORα expression in the interstitial tissue and epithelial cells compared with LSGs in normal controls. RORα detection may distinguish patients with and without pSS among patients with suspected pSS. However, it is necessary to define the criterion for quantifying RORα. Moreover, it is unknown whether there is a relationship between RORα and disease severity, as there is for the proportion of Th17 cells and IL-17 levels in peripheral blood lymphocytes from patients with pSS, and whether these diagnostic criteria are true in the salivary glands. If these criteria are also established in serum, it will be possible to diagnose pSS without performing an invasive lip biopsy. Since the sample size was small, the conclusion must be confirmed by further clinical case reports and experimental studies.

RORs can bind to certain small molecule ligands, such as cholesterol and oxysterols, to exert biological effects [41–43]. Recently, some synthetic ROR-specific inverse agonists have been reported and shown to be effective in controlling autoimmune diseases associated with Th17 cells in animal experiments [11, 44–48]. SR1001 is a highly selective and specific RORα/γt inverse agonist that specifically binds to RORα/γt and inhibits the differentiation of Th17 cells [44]. In our study, we investigated the effect of SR1001 in the treatment of pSS and found that SR1001 could significantly improve salivary gland function and alleviate lymphocyte infiltration into the submandibular glands; however, this effect was not obvious in the sublingual glands, since the submandibular glands in NOD mice are most likely to exhibit spontaneous sialadenitis [49]. Flow cytometry analysis showed that SR1001 could inhibit the differentiation of Th17 cells without affecting overall mouse development. In addition, SR1001 significantly controlled the progression of type I diabetes mellitus and had no obvious side effects on mouse development (Additional file 1). Older NOD mice had a greater improvement in salivary gland function after SR1001 treatment, so we speculated that it is possible that RORα has a different effect at different stages of pSS. At the early stage of pSS, RORα does not have a major effect because it has lower expression or activity, while at the progressive stage, it is prominent because of higher expression or activity. Although we showed that SR1001 can alleviate inflammation and improve salivary gland function, with better results in older NOD mice, additional studies are necessary to confirm this result.

Since SR1001 not only inhibits RORα, but also inhibits RORγt [44, 48], it was uncertain whether these effects stemmed from the inhibition of RORα and/or RORγt. We also detected the expression of RORγt to determine which is dominant in the process of inflammation of the LSGs. The results showed that the expression of RORγt in the LSGs in patients with pSS was markedly higher than that in patients without pSS (Additional file 2A, B). RORγt-positive cells were seen in infiltrated lymphocytic cells and also in some acinar cells and ductal cells on immunohistochemical staining (Additional file 2C). However, we realized that the expression of RORγt displayed no obvious increasing tendency with progressive disease stage/FS, and RORγt overexpression was even more evident in scattered lymphocytes from the LSGs of patients with pSS, who had a lower histological score for inflammatory lesions. This was different from what we found about RORα in the present study. Based on this finding, we speculated that acinar cells or salivary gland duct cells themselves could have RORγt overexpression in pSS. It has been reported that RORγt overexpression induced severed spontaneous sialadenitis-like SS via RORγt overexpressed CD4+ cells and reduced Treg [13]. We hypothesized that this expression of RORγt by acinar cells or salivary gland duct cells may not be important in the differentiation of Th17 cells in patients with pSS. In addition, we did not find other data on representing relation between RORγt and pSS progression as what we found on RORα. Though several autoantigens have been identified in pSS, none of them are only pSS-specific. Data from our study might provide support to the notion that RORα may be an index sign of accessory diagnosis and classification for pSS. Nevertheless, RORα affects a wide range of biological processes, such as biological rhythms, so further studies are needed to validate potential underlying mechanisms of how RORα is involved in the progression of salivary gland inflammation in patients with pSS.

## Conclusion

In summary, this is the first study of different RORα expression between patients with pSS and normal controls through the effect on Th17 cells. In patients with pSS, RORα expression increased with increasing FS, and there was positive correlation between these two factors. Specific inhibition of RORα inhibited the differentiation of Th17 cells, relieved salivary gland inflammation and improved salivary gland function. Our study may offer a new prospect for the diagnosis, classification, and treatment of pSS.

## Additional file

Additional file 1: The effects of SR1001 on blood glucose level and weight gain in NOD mice. **A** Body weight gain in NOD mice treated with SR1001 or vehicle for 4 weeks. There were no significant differences among these groups. Data represent means ± SEM (*n* = 5 for each group). *p* > 0.05. **B** SR1001 ameliorated the blood glucose level in NOD mice at 8 weeks of age (***p < 0.001), whereas there was no obvious effect on 4-week old mice (*p* > 0.05). Data represent means ± SEM (*n* = 5 for each group). (JPG 453 kb)

Additional file 2: RORγt expression in LSGs. **A** Western bolt showed that expression of RORγt (56 kD) was also significantly higher in whole LSGs from patients with pSS (*n* = 6) compared to non-pSS (*n* = 4). **B** Gray scale analysis. Data were normalized for GAPDH. Result represent the mean ± SEM. ***p < 0.001. **C** Relative expression of RORγt messenger RNA (RNA) in whole LSGs from patients with pSS (*n* = 6) was significantly more abundant compared to normal controls (NC, *n* = 4) normalized for GAPDH mRNA. The mean ± SEM is shown. ***p < 0.001. **D** Immunohistochemical analysis of RORγt in pSS (*n* = 34) and normal (*n* = 10) LSGs. RORγt-positive cells are more numerous in LSGs of patients with pSS with different FS compared with normal controls. Scale bars = 50 μm; insets, × 2 magnification. (JPG 1351 kb)

## Abbreviations

ANA: Anti-nuclear antibody
BSA: Bovine serum albumin
C3: Component 3
C4: Component 4
CLNs: Cervical lymph nodes
DAPI: Diamidino-phenyl-indole
DMSO: Dimethyl Sulphoxide
ECL: Enhanced chemiluminescence
ESR: Erythrocyte sedimentation rate
FS: Focus score
GAPDH: Glyceraldehyde-3-phosphate dehydrogenase
GM-CSF: Granulocyte-macrophage colony stimulating factor
H&E: Hematoxylin and eosin
IgG: Immunoglobulin G
IL-17: Interleukin 17
IL-17RA: Interlerkin-17 receptor A
IκBζ: Nuclear factor κB inhibitor ζ
LSG: Labial salivary gland
MLNs: Mesenteric lymph nodes
MSG: Minor salivary gland
NC: Normal control
NOD: Non-obese diabetic
PBS: Phosphate-buffered saline
PCR: Polymerase chain reaction
PMA: Phorbol-12-myristate-13-acetate
pSS: Primary Sjögren’s syndrome
RF: Rheumatoid factor
RIPA: Radio-immunoprecipitation assay
RORα: Orphan nuclear receptors retinoic acid-related receptor α
RORγt: Orphan nuclear receptors retinoic acid-related receptor γt
SLG: Sublingual gland
SMG: Submandibular gland
SS: Sjögren’s syndrome
SSA: Anti-Sjögren’s-syndrome-related antigen antibody A
SSB: Anti-Sjögren’s-syndrome-related antigen antibody B
TBST: Tris-buffered-saline Tween
Th17: T helper 17
Treg: Regulatory T
