# Supplementary figures and images for: The role of RORα in salivary gland lesions in patients with primary Sjögren’s syndrome
Source: Arthritis Res Ther. 2018 Sep 6;20:205. doi: 10.1186/s13075-018-1698-5 (PMC6127992; doi:10.1186/s13075-018-1698-5)

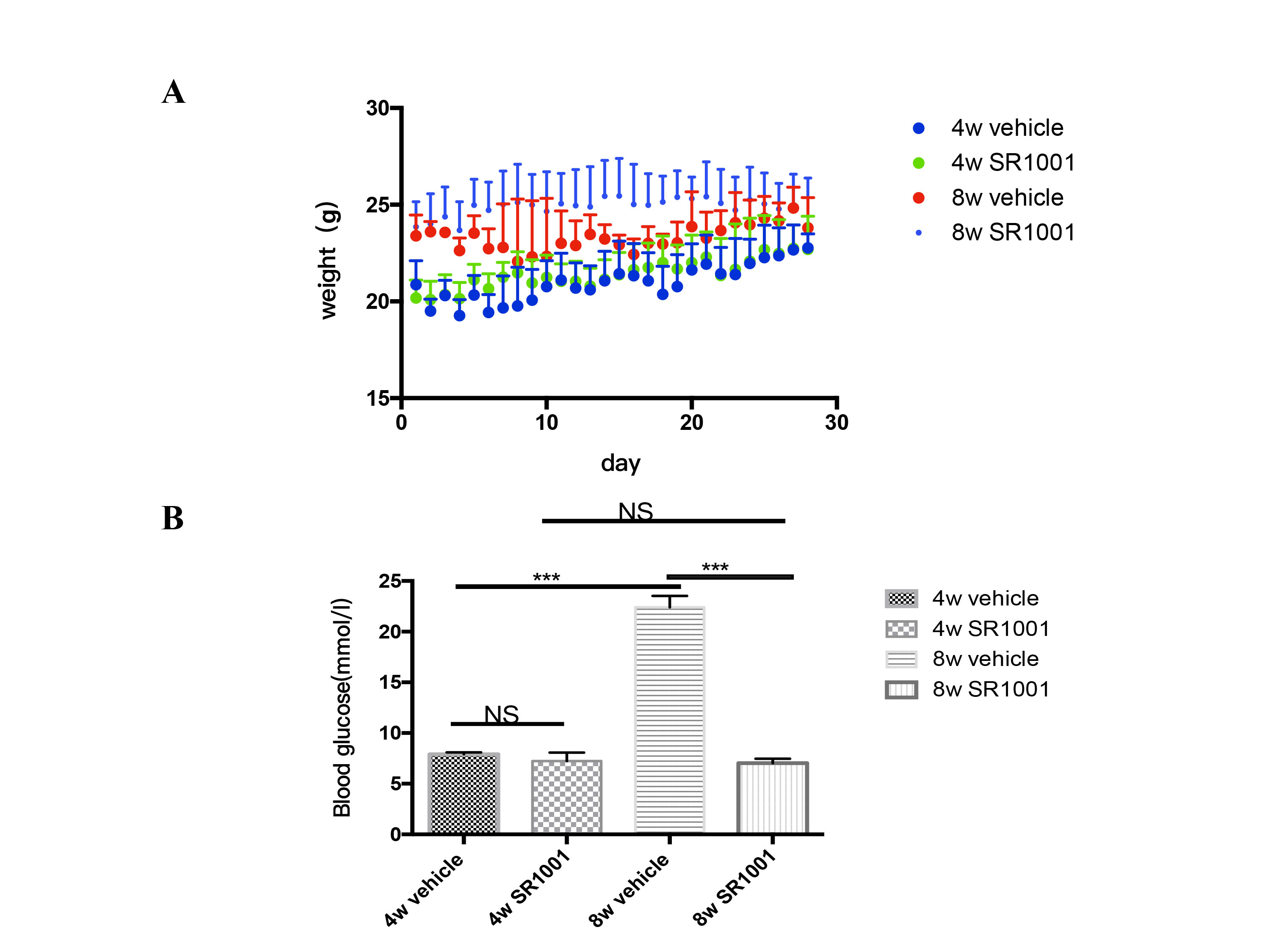

Supplement: Supplementary file 1 — The effects of SR1001 on blood glucose level and weight gain in NOD mice. A Body weight gain in NOD mice treated with SR1001 or vehicle for 4 weeks. There were no significant differences among these groups. Data represent means ± SEM (n = 5 for each group). p > 0.05. B SR1001 ameliorated the blood glucose level in NOD mice at 8 weeks of age (***p < 0.001), whereas there was no obvious effect on 4-week old mice (p > 0.05). Data represent means ± SEM (n = 5 for each group). (JPG 453 kb) [file 13075_2018_1698_MOESM1_ESM.jpg]

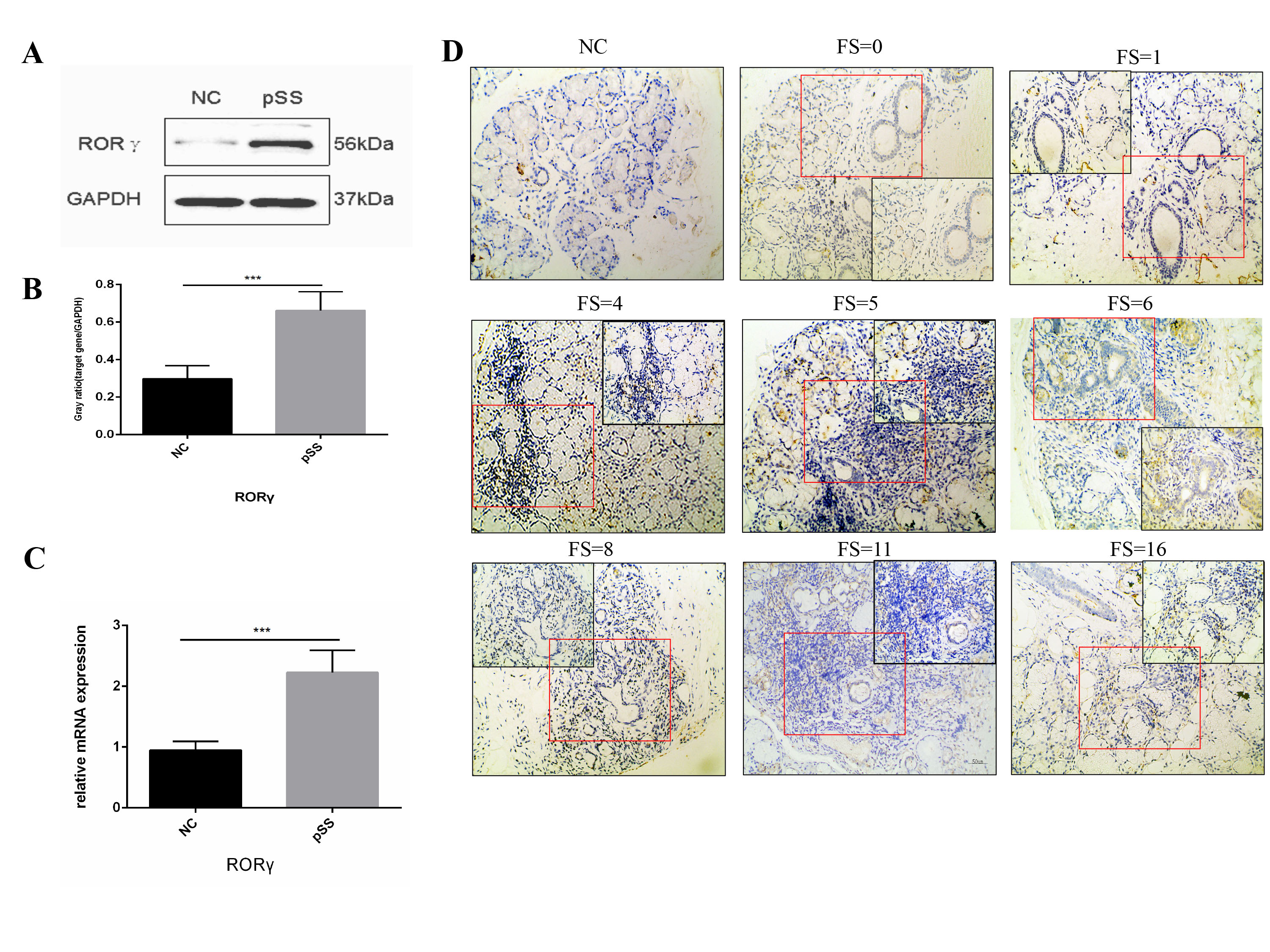

Supplement: Supplementary file 2 — RORγt expression in LSGs. A Western bolt showed that expression of RORγt (56 kD) was also significantly higher in whole LSGs from patients with pSS (n = 6) compared to non-pSS (n = 4). B Gray scale analysis. Data were normalized for GAPDH. Result represent the mean ± SEM. ***p < 0.001. C Relative expression of RORγt messenger RNA (RNA) in whole LSGs from patients with pSS (n = 6) was significantly more abundant compared to normal controls (NC, n = 4) normalized for GAPDH mRNA. The mean ± SEM is shown. ***p < 0.001. D Immunohistochemical analysis of RORγt in pSS (n = 34) and normal (n = 10) LSGs. RORγt-positive cells are more numerous in LSGs of patients with pSS with different FS compared with normal controls. Scale bars = 50 μm; insets, × 2 magnification. (JPG 1351 kb) [file 13075_2018_1698_MOESM2_ESM.jpg]
